# Biomarkers in heart failure with preserved ejection fraction

**DOI:** 10.1007/s12471-016-0817-7

**Published:** 2016-03-04

**Authors:** W. C. Meijers, A. R. van der Velde, R. A. de Boer

**Affiliations:** Department of Cardiology, University Medical Center, University of Groningen, Groningen, The Netherlands

**Keywords:** Heart failure, Preserved ejection fraction, Biomarkers, Natriuretic peptides, Diagnosis, Prognosis

## Abstract

Biomarkers are widely used and studied in heart failure. Most studies have described the utility and performance of biomarkers in sub-studies of randomised clinical trials, where the vast majority of the patients suffered from heart failure with reduced ejection fraction (HFrEF), and not with preserved ejection fraction (HFpEF). As a result, there is a scarcity of data describing the levels, dynamics, clinical and biochemical correlates, and biology of biomarkers in patients suffering from HFpEF, whereas HFpEF is in fact a very frequent clinical entity. This article discusses the value of different biomarkers in HFpEF. We describe various aspects of natriuretic peptide measurements in HFpEF patients, with a focus on diagnosis, prognosis and the risk prediction of developing heart failure. Further, we will discuss several emerging biomarkers such as galectin-3 and suppression of tumorigenicity 2, and recently discovered ones such as growth differentiation factor-15 and syndecan-1.

## Introduction

In the last decades we have seen an explosion in numbers of new and emerging biomarkers, and the number of articles on this topic has steadily risen [1–3]. There are several comprehensive review articles that describe the analytical issues, as well as the sensitivity and specificity, and other articles that present the performance of various biomarkers in various settings [4–6]. However, traditionally, heart failure was considered a disease characterised by contractile dysfunction and, as a result, most data on biomarkers were collected in patient cohorts with heart failure with reduced ejection fraction (HFrEF). Nowadays, we know that many patients suffer from heart failure with preserved ejection fraction (HFpEF), more related to a filling problem of the left ventricle but, importantly, also resulting in a pumping failure of the heart.

The aim of this overview is to describe several generally accepted and several novel biomarkers in HFpEF, focusing on the value these biomarkers have for diagnostic and prognostic performance, and potential clinical utility.

## Natriuretic peptides

The natriuretic peptides are by far the best studied and most widely accepted and employed biomarkers in heart failure, both in HFrEF and in HFpEF. The best studied natriuretic peptides are atrial natriuretic peptide and B-type natriuretic peptide (BNP). Mechanistically, transcription and release of both natriuretic peptides occurs in response to myocardial stretch [7, 8]. Besides BNP, clinicians can also measure NT-pro B-type natriuretic peptide (NT-proBNP), which is the biologically inactive fragment that is formed after cleaving proBNP into the active hormone BNP (ratio 1:1) [7–9].

Nowadays, natriuretic peptides can easily be measured by fully automated and commercially available assays, which have proven excellent precision and reproducibility, also between laboratories. BNP and NT-proBNP correlate very well, but the clearance of NT-proBNP is exclusively via the kidney whereas BNP is also cleared via the liver, resulting in different half-lives of the hormones.

### Natriuretic peptides in HFpEF

**Fig. 1:**
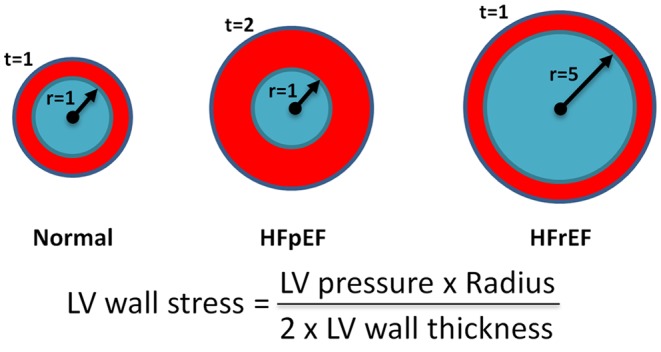
The law of Laplace

Natriuretic peptides are moderately elevated in HFpEF patients [10–14], and levels may drop down to normal or near normal in symptom-free periods. This can be explained since natriuretic peptides are released and produced in response to increased myocardial wall stress. HFpEF is characterised by hypertrophic hearts with a small left ventricular (LV) cavity, and this structural abnormality in itself does not elevate end-diastolic wall stress much, as can perfectly be concluded from Laplace’s law (Fig. 1). Under specific circumstances, such as supraventricular tachycardia or fluid overload, levels of natriuretic peptides may become very high (as in HFrEF), but this is uncommon. The lack of chronic elevations in wall stress comprehensively results in less natriuretic peptide production and lower circulating levels when compared with the levels in HFrEF. However, although the plasma levels of natriuretic peptides do not show extreme increases, it has been demonstrated that they gradually rise in parallel to the severity of diastolic abnormalities as assessed by e.g. echocardiography [10–14].

### Identification of chronic stable HFpEF patients

There are no established cut-off points for HFpEF versus HFrEF, or versus heart failure as an entire group.

According to the heart failure guidelines [15] natriuretic peptides may be measured in the outpatient clinic to help stratify patients who are suspected for a diagnosis of heart failure (regardless of whether this HFpEF or HFrEF), and for whom it may be useful to order echocardiography. The primary goal is to distinguish between symptomatic (dyspnoeic) patients, who do or do not have heart failure. Exclusionary cut-off points can be applied to this aim, because of very high negative predictive values (very low risk of false negatives). The combination of the medical history, signs and symptoms, and natriuretic peptide levels may provide the clinician with a very good feel as to whether or not heart failure exists, and echocardiography will then lead to the diagnosis of heart failure, and can directly differentiate between HFpEF or HFrEF. The European Society of Cardiology (ESC) guidelines on heart failure propose a cut-off of 125 pg/ml for NT-proBNP and 35 pg/ml for BNP in the above-described setting [15].

Tschöpe et al. conducted a study that provided evidence that natriuretic peptides may be used to diagnose HFpEF. Herein, the authors demonstrated that NT-proBNP levels were strongly related to increased LV diastolic filling pressures, as determined by invasive measurements and end-diastolic wall stress in HFpEF patients [13]. The latter was confirmed by others [14], and NT-proBNP levels also appear to be related to tissue remodelling and fibrosis formation, which might also play a pivotal role in impaired relaxation. However, without exception these patients were ‘pre-selected’, in the sense that they were referred because of a clinical suspicion of heart failure, and therefore had a high likelihood of having HFpEF, so that the cut-off points derived from these studies cannot be applied to all comers or asymptomatic subjects.

### Identification of acute HFpEF patients

Clearly, acute heart failure patients present completely differently than chronic stable heart failure patients. A crucial difference is that patients present acutely, usually to the emergency department, with symptoms of dyspnoea. During such episodes, the cardiac muscle endures high wall stress, which results in high values of natriuretic peptides in those with acute heart failure, regardless of a preserved or reduced ejection fraction. Maisel et al. showed that HFpEF patients who present with acute decompensated heart failure typically had BNP values of 600–1000 pg/ml [11]. Less severe HFpEF, in patients who were more compensated, resulted in lower BNP values (between 100 and 600 pg/ml) [16]. In a recent systematic review and meta-analysis that included 37 different study cohorts, the exclusionary cut-point (for overall heart failure) of 100 pg/ml was confirmed for BNP and 300 pg/ml for NT-proBNP [17].

### Prediction of new-onset HFpEF in the general population

Besides their diagnostic utility, natriuretic peptides might be used to identify subjects at risk of developing HFpEF over the course of many years, which is different from the diagnostic utility of natriuretic peptides. A possible utility for ‘signalling’ heart failure over a very long course has been shown for natriuretic peptides, and high-sensitivity troponin, for new-onset HFpEF in high-risk subjects from the general population [18], and the elderly [19]. Previously, natriuretic peptides have been shown to be associated with preclinical diastolic dysfunction and the propensity to develop heart failure [20], although the performance of natriuretic peptides for the detection of subclinical diastolic dysfunction is not as good as for symptomatic diastolic dysfunction [21]. Therefore, natriuretic peptides alone lack the prognostic power to identify these patients, and it seems reasonable that a combination with clinical, electrocardiographic, and biochemical risk factors may comprise a useful and powerful model for detecting those at risk for developing HFpEF in the community.

### Plasma biomarkers: predicting prognosis in HFpEF

Epidemiological studies suggest that the overall prognosis is comparable for patients with HFpEF compared with HFrEF. However, in randomised controlled trials (RCTs), a certain bias exists because a ‘typical’ HFpEF patient usually has multiple comorbidities and often these comorbidities are exclusion criteria for trials, and as a result patients suffering from them are excluded from such trials. Therefore, the patients with a high likelihood for an adverse event are paradoxically often excluded from HFpEF trials, and this results in a substantially lower cardiovascular risk in HFpEF patients enrolled in RCTs than in HFrEF patients enrolled in RCTs.

Nevertheless, the i-PRESERVE and the PEP-CHF studies demonstrated that both baseline values of NT-proBNP and change in NT-proBNP from baseline have prognostic value in patients with HFpEF, improving the prediction of mortality and heart failure rehospitalisation [22, 23]. When NT-proBNP increases from the baseline value, this was associated with an increased mortality and morbidity, while decreases in NT-proBNP levels were associated with reduced mortality and morbidity rates [22]. Van Veldhuisen et al. showed that despite the fact that natriuretic peptide levels are lower in HFpEF patients, compared with HFrEF patients, the predictive value of a given value of NT-proBNP is equal regardless of the ejection fraction [24].

### Comorbidities common in HFpEF and their influence on natriuretic peptide levels

Atrial fibrillation is very common in HFpEF, and the presence of atrial fibrillation has a strong impact on circulating levels of natriuretic peptides. In patient cohorts with paroxysmal and chronic atrial fibrillation, natriuretic peptide levels are strongly elevated, often exceeding the values reported in HFpEF [25]. McKelvie et al. [26] even described a fivefold increase of natriuretic peptides compared with HFpEF patients with atrial fibrillation compared with HFpEF patients in sinus rhythm. Therefore, natriuretic peptide levels in HFpEF patients with atrial fibrillation should be considered differently to the levels in HFpEF patients in sinus rhythm.

Besides atrial fibrillation, female sex and advanced age, both very common in HFpEF, are associated with elevated NT-proBNP [27]. In a study by McCullough et al. renal function was a confounder of BNP levels, especially in those with an eGFR less than 60 ml/min/1.73 m^2^. Finally, obesity is associated with lower BNP levels [28, 29], and lower cut-off values may be considered once the body mass index exceeds 35 kg/m^2^. Therefore clinicians who treat HFpEF patients and measure natriuretic peptide levels are advised to take the above-mentioned comorbidities into consideration when assessing the natriuretic peptide values.

## Galectin-3 and suppression of tumorigenicity 2

Galectin-3 and suppression of tumorigenicity 2 (ST2) are emerging biomarkers that are not only predictive for hospitalisation and death in patients with heart failure, but also add additional prognostic value over natriuretic peptides. As such, they received a class IIB recommendation by the 2013 American College of Cardiology/American Heart Association guideline for the management of heart failure for risk stratification [30].

Evidence has been generated specifically regarding the prognostic value of galectin-3 and ST2 in HFpEF patients, and patients with preclinical diastolic dysfunction.

### Galectin-3

One of the first studies that compared galectin-3 levels between HFpEF and HFrEF patients was conducted by de Boer et al. [31]. The Coordinating study evaluating Outcomes of Advising and Counseling in Heart Failure (COACH) enrolled patients at discharge after being admitted for acute heart failure, and 20 % (107 patients) were diagnosed with HFpEF (LVEF > 40 %). Interestingly, the authors found that galectin-3 appeared to have a particularly strong predictive value in HFpEF patients, compared with HFrEF patients. This finding highlighted the possibility of different pathophysiological mechanisms in the two sub-types of heart failure.

Three additional HFpEF cohorts have since demonstrated the relation between galectin-3 and HFpEF.

First, Carrasco-Sánchez and colleagues demonstrated the predictive value of galectin-3 in 419 HFpEF patients (LVEF > 45 %) who were admitted with acute heart failure. Galectin-3 independently predicted all-cause mortality and heart failure rehospitalisation, and yielded significant reclassification indices [32]. Currently this study is one of the largest HFpEF biomarker studies that has been published.

Secondly, the ALDO-HF trial randomised HFpEF (LVEF ≥ 50 %) patients to either a mineralocorticoid receptor antagonist (MRA, spironolactone) or placebo. Spironolactone improved LV diastolic function, but did not affect maximal exercise capacity, patient symptoms, or quality of life [33]. Galectin-3 levels were measured at baseline, 6 months and 12 months and were only modestly elevated at baseline (median 12.1 ng/ml). Nevertheless galectin-3, and specifically increases in galectin-3, were independent of treatment and NT-proBNP associated with all-cause mortality or heart failure rehospitalisation [34].

The RELAX trial [35] was a multicentre, double-blind, placebo-controlled, parallel-group, randomised clinical trial of 216 stable outpatients with HFpEF (LVEF ≥ 50 %), evaluating the efficacy of sildenafil or placebo for 24 weeks. The authors showed in this third study that galectin-3 levels were associated with age, smaller body size, and severity of renal dysfunction [36]. However, galectin-3 levels were not associated with comorbidities, symptomatic status or congestion, severity of LV remodelling or dysfunction, or exercise performance after adjusting for age, sex, and cystatin-C. It could be hypothesised that galectin-3, a biomarker associated with the remodelling process, may be less useful in patients with end-stage disease.

### ST2

The first evidence that ST2 levels could be of interest in HFpEF patients was provided by a post-hoc analysis of 200 HFpEF patients [37]. Although the study cohort consisted largely of African-Americans, ST2 was a better predictor for mortality than NT-proBNP. However, ST2 concentrations did not correlate with echocardiographic indices of LV diastolic function.

More clinical data on ST2 in HFpEF was provided by Friões et al. [38]. The authors divided patients with acute heart failure based on LVEF, and reported that NT-proBNP predicted all-cause mortality or heart failure readmissions at 6 months regardless of LVEF. ST2 was reported to be a significant predictor of prognosis in HFrEF patients, but not in HFpEF patients. In contrast to the above-mentioned findings, data were reported of a pooled analysis including three cohorts from Boston, Massachusetts, Linz, Austria and Murcia, Spain. In 447 HFpEF patients admitted for acute heart failure, the authors demonstrated a comparable prognostic value of ST2 in both HFpEF and HFrEF [39].

Hypertension, a comorbidity that is most frequently present in HFpEF patients, might lead to different activated pathophysiological mechanisms in HFpEF. This was recently investigated by Zile et al. [40]. The authors measured ST2 in 70 coronary artery bypass graft patients stratified into three groups, control (no hypertension), hypertensive patients without HFpEF and hypertensive patients with HFpEF. These patients underwent echocardiography, biomarker assessment, and LV epicardial anterior wall biopsy. ST2 was higher in the hypertensive patients without HFpEF compared with the control subjects. The ST2 levels further increased in the hypertensive patients with HFpEF. Besides these increasing levels, ST2 was significantly correlated with pulmonary capillary wedge pressure and increased collagen-dependent stiffness. These findings support the hypothesis that ST2 is involved in HFpEF development and might play an early role in the induced pro-inflammatory pro-fibrotic state due to hypertension.

## Other biomarkers in HFpEF

**Fig. 2:**
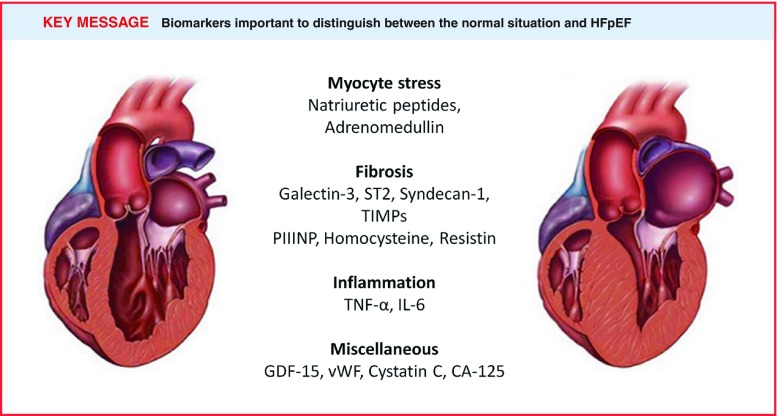
Circulating biomarkers associated with pathophysiology of HFpEF. Since HFpEF is such a heterogeneous disease, it comes as no surprise that biomarkers which reflect various domains of the disease (myocardial structural remodelling and stretch, inflammation, fibrosis, kidney function and more) are increased and may be used for the diagnosis and prognosis of HFpEF. Adapted from Mayo Foundation for Medical Education and Research

Besides natriuretic peptides, galectin-3 and ST2 several other biomarkers have been identified as potential markers for HFpEF. With respect to the biomarker levels, tissue inhibitor of metalloproteinases (TIMPs), aminoterminal propeptide of type III procollagen (PIIINP), homocysteine and resistin are particularly upregulated in HFpEF compared with HFrEF. Whereas biomarkers of inflammation, such as pentraxin-3, C-reactive protein, tumour necrosis factor alpha (TNF-α), interleukin (IL)-6 and IL-10, show higher levels in HFrEF compared with HFpEF (Fig. 2).

### Other markers of myocyte stress

Whereas NT-proBNP is the gold standard for myocyte stress, there are also other markers of cardiac loading that seem to have value in HFpEF. One of such factors is adrenomedullin, which is a hormone that is able to decrease systemic vascular resistance and also has diuretic and natriuretic effects. Levels of this hormone are elevated in patients with HFrEF, compared with healthy controls and identify patients with a restrictive filling pattern [41]. An additional diagnostic or prognostic value over NT-proBNP has not been demonstrated.

### Other markers of extracellular matrix remodelling

Interstitial fibrosis is an important feature of heart failure with preserved ejection fraction, but myocyte stiffness plays an even more important role. This is reflected by the level of markers of extracellular matrix remodelling, such as matrix metalloproteinases (MMPs) 2 and 9, carboxy-terminal telopeptide of collagen type I (CITP) and PIIINP. All these biomarkers are associated with outcome in HFpEF, although the independent predictive value of each marker was limited [42]. Syndecan-1 is a new marker of fibrosis and a member of the proteoglycan family. Its levels were correlated with other fibrotic biomarkers and were associated with clinical outcome in HFpEF, but not in HFrEF. Syndecan-1 also showed improvement of risk stratification on top of clinical risk factors, including NT-proBNP [43].

### Markers of inflammation

Inflammatory markers play an important role in the development of heart failure. TNF-α and IL-6 were both associated with new-onset HFpEF, but less associated with HFrEF [44].

### Other markers

Next to markers of myocyte stress, inflammation and extracellular matrix remodelling, also other biomarkers are studied for their value in HFpEF. These biomarkers include growth differentiation factor-15 (GDF-15), cystatin C, resistin, cancer antigen-125 (CA-125) and von Willebrand factor (vWF).

GDF-15 is a member of the transforming growth factor (TGF)-beta family and correlates with cardiac hypertrophy and fibrosis. In 149 patients with LV diastolic dysfunction and normal ejection fraction, GDF-15 was found to be a potentially useful prognostic biomarker in patients with HFpEF, although the independent value was not assessed [45]. However, GDF-15 is not specific for HFpEF, it is also an independent predictor of mortality in patients with HFrEF [46].

Cystatin C is a marker of renal function that also predicts cardiovascular outcome. The serum cystatin C level in patients with HFpEF is an independent predictor for all-cause mortality and/or readmission in patients with acute heart failure, regardless of renal function [47]. However, the prognostic value of cystatin C is lower in patients with HFpEF, compared with HFrEF patients [48].

Resistin is derived from adipose tissue and associated with inflammation. Resistin was measured in 2902 subjects in the Health ABC study and was associated with incident HFpEF and to a lesser extent with HFrEF. The prognostic value is yet to be examined [49].

CA-125 is a tumour marker for ovarian cancer, but is also elevated in heart failure and is related to the severity of heart failure [50]. CA-125 levels increase when the heart failure stage or the level of diastolic dysfunction is more severe, although the levels of CA-125 were not different between patients with HFpEF and control patients [50].

vWF is an independent predictor of long-term outcome in patients with HFpEF, which may be explained by its role in endothelial dysfunction [51]. vWF was measured in 457 patients of the Ludwigshafen Risk and Cardiovascular Health study and showed additional value beyond NT-proBNP [51].

## Conclusions

A large proportion of patients with heart failure suffer from HFpEF. In contrast to HFrEF, the role for biomarker assessment in HFpEF has not been validated in full extent. No biomarker is specific for HFpEF; however, several markers have been shown to provide powerful and clinically meaningful information for the setting of the diagnosis and prognosis of HFpEF. As HFpEF is a very heterogeneous syndrome, likely not one but rather a panel of various biomarkers, representing different aspects of the pathophysiology, will provide the most powerful and sophisticated information for clinicians caring for patients with HFpEF. In the coming decades, such panels need to be identified, validated, and prospectively tested in the outpatient and inpatient setting.
